# Identification of Partial Discharge Sources by Feature Extraction from a Signal Conditioning System

**DOI:** 10.3390/s24072226

**Published:** 2024-03-30

**Authors:** Itaiara Felix Carvalho, Edson Guedes da Costa, Luiz Augusto Medeiros Martins Nobrega, Allan David da Costa Silva

**Affiliations:** Department of Electrical Engineering, Federal University of Campina Grande, Aprigio Veloso 882, Universitário, Campina Grande 58429-900, Brazil; edson@dee.ufcg.edu.br (E.G.d.C.); allan.costa@ee.ufcg.edu.br (A.D.d.C.S.)

**Keywords:** signal conditioning system, partial discharge, classification of partial discharges

## Abstract

This paper addresses the critical challenge of detecting, separating, and classifying partial discharges in substations. It proposes two solutions: the first involves developing a signal conditioning system to reduce the sampling requirements for PD detection and increase the signal-to-noise ratio. The second approach uses machine learning techniques to separate and classify PD based on features extracted from the conditioned signal. Three clustering algorithms (K-means, Gaussian Mixture Model (GMM), and Mean-shift) and the Support Vector Machine (SVM) method were used for signal separation and classification. The proposed system effectively reduced high-frequency components up to 50 MHz, improved the signal-to-noise ratio, and effectively separated different sources of partial discharges without losing relevant information. An accuracy of up to 93% was achieved in classifying the partial discharge sources. The successful implementation of the signal conditioning system and the machine learning-based signal separation approach opens avenues for more economical, scalable, and reliable PD monitoring systems.

## 1. Introduction

Electrical power equipment is subject to electrical, mechanical, thermal, and environmental stresses. These stresses can impact the insulation either individually or in combination. Electrical insulation in high-voltage equipment is one of the main failure factors [1,2,3]. Degradation of the insulation may be associated with the continuous action of partial discharges (PDs).

PDs are low-intensity electrical discharges that partially short-circuit regions of the insulating material subjected to intense electric fields [4]. Continuous PD activity gradually degrades the insulation system, which can lead to the failure of high-voltage electrical equipment. Therefore, PD detection and interpretation are robust tools for condition monitoring and risk assessment of insulation failures in high-voltage equipment [5,6,7].

Traditionally, PDs are detected according to the procedures described by the method included in the international standard [8]. This method requires the use of a coupling capacitor connected in parallel with the monitored equipment. Due to the invasive nature of using the coupling capacitor, the level of noise present in a substation, and the complexity of determining the real generating source of PD, this method is mostly used in the laboratory.

As alternatives, other methods have been developed for monitoring PD or its effects in substations, including dissolved gas analysis (DGA) [9,10], the measurement method via High-Frequency Current Transformers (HFCTs) [11,12], the acoustic detection method [13,14], and the radiometric method [15,16,17,18].

Radiometric detection is based on the principle of detecting electromagnetic waves by antennas [19]. Thus, the radiometric method has the advantages of having a non-invasive nature, sensitivity in the desired frequency range, and robustness in the face of corona discharge signals, whose significant components are comprised in frequency ranges of up to 300 MHz [20,21,22]. Initially, the radiometric method was applied to monitor a gas-insulated substation (GIS) [19,23,24,25,26]. With the consolidation of the radiometric method for the detection, classification, and localization of PD in GIS, researchers expanded their studies to the application of the radiometric method in other high-voltage equipment, such as power transformers [27,28,29,30,31]. Thus, the radiometric method established itself as a method implemented in the detection, location, and classification of PD in electrical equipment [15,16,27,28].

The classification of PD sources is commonly performed using Phase-Resolved Partial Discharge (PRPD) analysis. By PRPD analysis, it is possible to observe, in a single graph, the occurrences of PD in a specific phase of the applied voltage, with a certain magnitude of charge. One of the main challenges of using PRPD for the classification process, and subsequent diagnosis, is the simultaneous presence of multiple sources of PD or noise. The patterns obtained under these conditions are difficult to interpret, because the signals can overlap, and higher-amplitude sources can hide the presence of other types of sources with lower amplitudes [29,32,33]. For this reason, it is recommended to perform a separation process to assist in defect identification [33,34,35].

**Figure 1:**
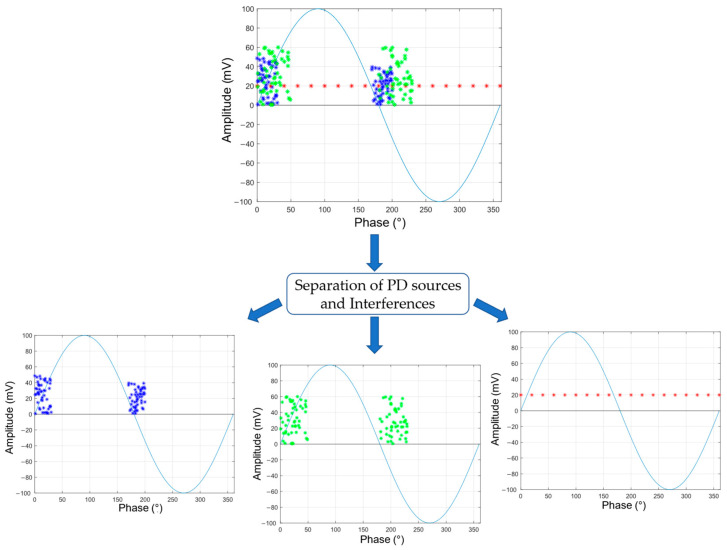
Separation of PD sources (blue and green signals) and interference (red signal).

In Figure 1, an example of overlapping sources of PD (blue and green signals) and interference (red signal) in a PRPD graph can be observed. As shown in Figure 1, the step of separating the PD sources should be performed before interpretation to correctly identify each source and avoid omitting potential associated defects. By separating the signals, it becomes possible to distinguish and analyze each one individually, preventing relevant information from being overlooked. This approach allows the identification of equipment with discharges and contributes to their proper maintenance and monitoring.

The detection, location, and classification of PD using the radiometric method require PD signals to be obtained in the ultra-high-frequency (UHF) spectrum. Therefore, it is necessary to use acquisition systems with a high sampling rate. High sampling rate acquisition requires proper instrumentation and accessories, such as oscilloscopes, spectrum analyzers, cables, and connections for UHF. However, in substation environments, there are intense electric and magnetic fields, as well as adverse environmental conditions such as humidity, pollution, and temperature variations. Under these conditions, the performance and durability of the instruments used may be compromised. Additionally, there is a high risk of equipment failure due to the presence of electric and magnetic fields, as well as the occurrence of surges. Therefore, it is safer and more efficient to use dedicated monitoring systems, specially designed for online and real-time detection and monitoring of PD in substations. Alternative techniques [36,37,38] have been developed to decrease the hardware requirements of the monitoring system using the PD radiometric signal envelope. However, research in this field is still in its early stages, and there is a need to develop a system that can reduce the sampling requirements while retaining important information from the PD signal.

This paper proposes complementary solutions. The first proposed solution is the development of a signal conditioning system, based on the envelope detection technique, which reduces the hardware requirements of the monitoring system while preserving relevant signal information. This approach aims to increase the applicability of the method in substations, making it more accessible and reliable.

The second proposed solution involves the use of information extracted from the signal envelope to perform signal separation, using machine learning techniques. This approach makes it possible to accurately distinguish different sources of PD and noise to assist in defect identification.

Therefore, the main purpose of this paper is to develop a signal conditioning system that reduces the sampling rate requirements and can be used in the detection, localization, and classification of PD signals, as well as to develop dedicated algorithms for the identification and separation of PD sources. The system is based on obtaining the envelope of the PD signal and using extracted features from the envelope for the classification, identification, and separation of PD sources.

## 2. Signal Conditioning System

The signal conditioning system is based on obtaining the envelope of the PD signal and using extracted features from this envelope for the classification, identification, and separation of PD sources and interference. Therefore, the system has been designed to capture signals in the UHF spectrum and generate envelopes with reduced frequency (up to 100 MHz) while preserving sufficient information for the analysis of the PD signals. 

**Figure 2:**
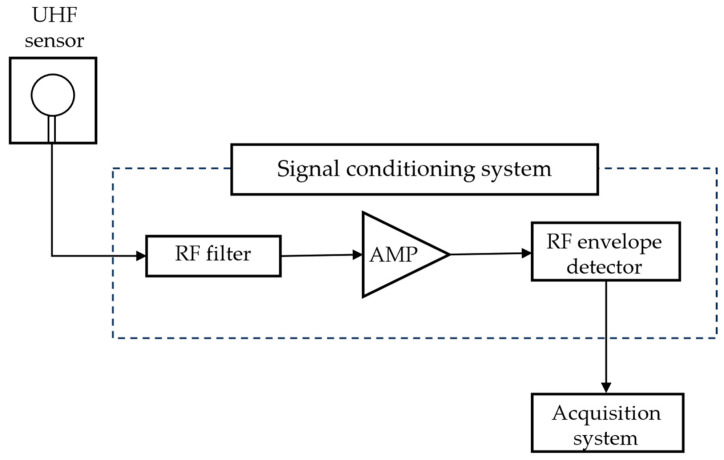
Schematic of PD detection with signal conditioning system.

Figure 2 shows the schematic of PD detection with the signal conditioning system.

The signal emitted by the test object is detected by the UHF sensor in series, with the signal conditioning system composed of a high-pass filter, an RF amplifier, and a sensing board with envelope detection.

The RF high-pass filter applied has a cutoff frequency of 100 MHz and a Butterworth response and is a seventh-order filter. To increase the sensitivity in detecting PD signals, the RF amplifier with a frequency range of 30 MHz to 4000 MHz (40 dB) was used. Finally, to detect signals in the UHF spectrum, an envelope detector with a suitable non-linear device, a Schottky diode, was used. The envelope detector circuit can operate from DC to 6 GHz on signals with an envelope bandwidth of up to 130 MHz.

**Figure 3:**
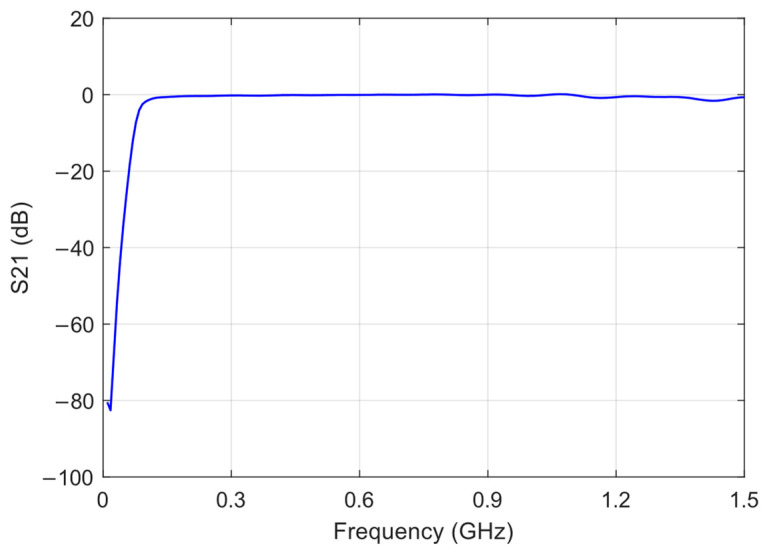
Magnitude of the S_21_ transmission coefficient of the RF high-pass filter.

**Figure 4:**
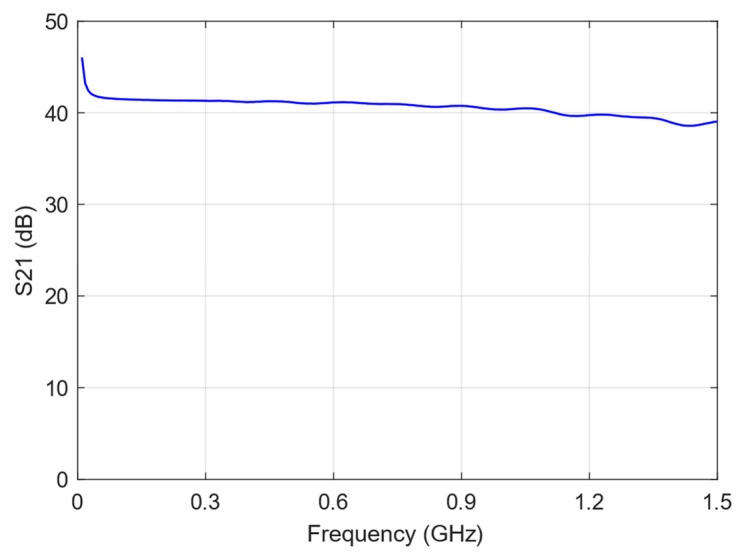
Magnitude of the S_21_ transmission coefficient of the RF amplifier.

**Figure 5:**
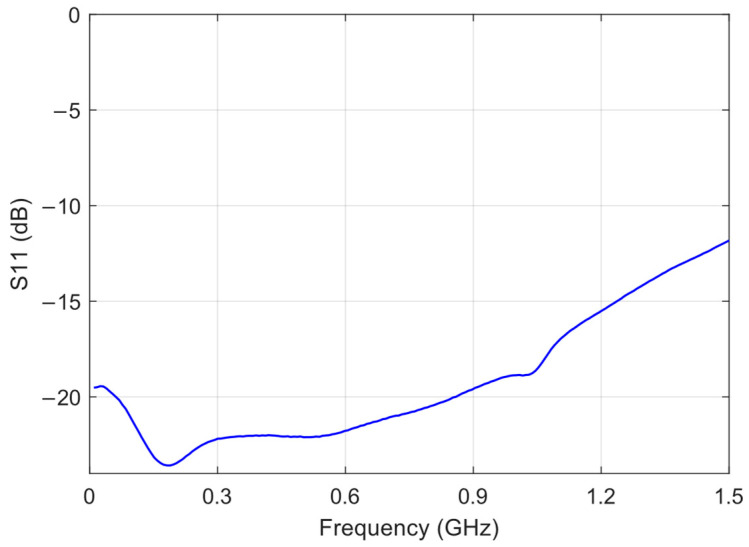
Magnitude of the S_11_ reflection coefficient of the sensing board with envelope detector.

The E5062A ENA RF Network Analyzer was used to measure the transmission coefficient, S_21_, of both the RF filter and the RF amplifier, as well as the reflection coefficient, S_11_, of the sensing board with the envelope detector. Figure 3, Figure 4 and Figure 5 show the graphs representing the magnitude of the S_21_ coefficient for the RF filter and amplifier, and the magnitude of the S_11_ coefficient for the envelope detector, respectively.

The S_21_ transmission coefficient indicates how the transmission line transmits the signal and is related to the insertion loss. As a result, the filter exhibits low insertion loss, less than 0.43 dB, within the frequency range of 300 MHz to 1500 MHz. The RF amplifier gain exceeds 40 dB up to a frequency of 1.1 GHz, with a minimum gain of 38.5 dB between 1.1 GHz and 1.5 GHz.

S_11_ represents the ratio of the power reflected from the envelope detector and the power delivered to the transmission line. The envelope detector presented reflection coefficients below −10 dB between 0.3 GHz and 1.5 GHz, proving to be suitable for detecting PD.

**Figure 6:**
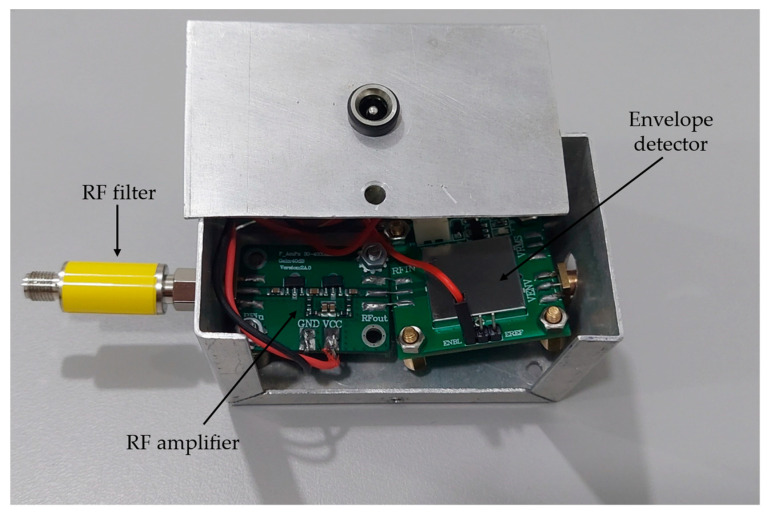
Photograph of signal conditioning system.

A photograph of the signal conditioning system is shown in Figure 6.

After carrying out the tests with the signal conditioning system applied in the experimental arrangement, the PD signal envelopes acquired from the three test objects were used for feature extraction, to be discussed in the next section.

## 3. Feature Extraction and Selection

### 3.1. Algorithm Proposed for Feature Extraction 

The process of feature extraction from the envelopes of PD signals was performed using algorithms. The PD envelope signals from different sources were used as the input to the algorithm, and a method for extracting basic and statistical features was implemented. At the end of the algorithm, basic quantities related to the signal waveform and statistical features were extracted from the envelopes, such as rise time (${\;T}_{r}$), fall time (${\;T}_{f}$), envelope time ($T_{t}$), signal energy (${\;E}_{s}$), peak value ($V_{p}$), root mean square value $\left(V_{RMS}\right)$, average amplitude $\left(A_{a}\right)$, square root amplitude ($A_{r2}$), crest factor ($C_{f}$), clearance factor (${CL}_{f}$), impulse factor ($I_{f}$), shape factor ($S_{f}$), skewness ($S_{k}$), and kurtosis ($K_{u}$).

Basic quantities can be defined as the intrinsic parameters of the PD pulse, such as signal intensity, rise time, pulse duration, and signal energy. However, statistical operators are operators for the statistical analysis of parameters derived from PD signals [39]. Among the main derived and statistical features, the following stand out:

- Peak value (*$V_{p}$*):
  (1) $$V_{p}\left(x\right)=max\left(x_{i}\right),\;i=1,\;2...N.$$
- Root mean square value ($V_{RMS}$):
  (2) $$V_{RMS}\left(x\right)=\sqrt{\frac{1}{N}\sum_{i=1}^{N}x_{i}^{2}}.$$
- Average amplitude ($A_{a}$):
  (3) $$A_{a}\left(x\right)=\frac{1}{N}\sum_{i=1}^{N}\left|x_{i}\right|.$$
- Square root amplitude ($A_{r2}$):
  (4) $$A_{r2}\left(x\right)={\left(\frac{1}{N}\sum_{i=1}^{N}\sqrt{\left|x_{i}\right|}\right)}^{2}.$$
- Crest factor ($C_{f}$):
  (5) $$C_{f}\left(x\right)=\frac{V_{p}\left(x\right)}{V_{RMS}\left(x\right)}.$$
- Clearance factor (${CL}_{f}$):
  (6) $${CL}_{f}\left(x\right)=\frac{V_{p}\left(x\right)}{A_{r2}\left(x\right)}.\;\;$$
- Impulse factor ($I_{f}$):
  (7) $$I_{f}\left(x\right)=\frac{V_{p}\left(x\right)}{A_{a}\left(x\right)}.\;\;\;$$
- Shape factor ($S_{f}$):
  (8) $$S_{f}\left(x\right)=\frac{V_{RMS}\left(x\right)}{A_{a}\left(x\right)}.\;\;$$
- Skewness ($S_{k}$):
  (9) $$S_{k}\left(x\right)=\frac{E{\left(x-\mu \right)}^{3}}{\sigma^{3}}.\;\;\;\;$$
- Kurtosis ($K_{u}$):
  (10) $$K_{u}\left(x\right)=\frac{E{\left(x-\mu \right)}^{3}}{\sigma^{4}}.\;$$
  where $x_{i}$ is the value of sample *i*, N is the total number of points, μ is the mean of x, σ is the standard deviation of x, and E represents the expected value.

### 3.2. Algorithm Proposed for Feature Selection

The feature selection was performed to investigate the quality of features in promoting the separation of PD sources into clusters, detecting similar patterns among different variables, and thereby improving the efficiency of the classification algorithm. In this paper, the features were selected based on the results provided by recursive feature elimination (RFE) [40], mutual information [41], and correlation analysis [34,36].

Initially, the data were normalized, ensuring that the selection methods operated fairly and effectively, regardless of the different original scales of the features. Next, the RFE and mutual information dimensionality reduction methods were applied.

The RFE method was applied with a multinomial logistic regression model. RFE operates by recursively selecting features in progressively smaller sets [40]. Initially, the model is trained on the complete set of features, and the importance of each feature is assessed. Subsequently, the less important features are removed, and this process is repeated recursively until the desired number of features is obtained.

The mutual information technique was also employed for feature selection. The mutual information technique calculates the entropy of the variables [41]. Thus, it determines the amount of shared information between the variable of interest (target value) and each feature.

The RFE and mutual information models were adjusted to select five features. The features selected were analyzed using correlation analysis to select less redundant features. This crucial step aims to ensure the choice of features that not only exhibit significant individual variability but are also complementary and less correlated with each other. 

**Figure 7:**
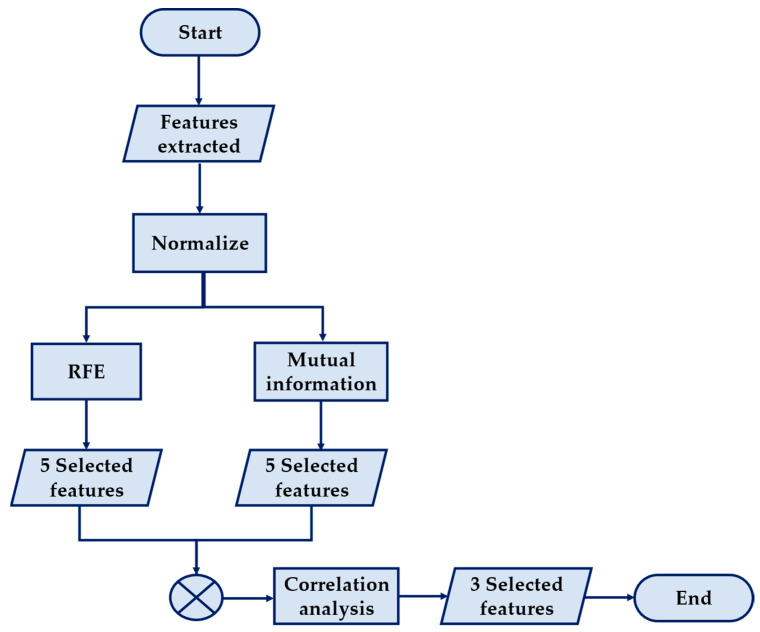
Feature selection algorithm flowchart.

The final number of features chosen was three, driven by two primary reasons. Firstly, limiting the selection to just one or two features would overly burden the model’s reliance on those attributes. Opting for three features enhances the model’s robustness, ensuring a more balanced separation and classification. Additionally, the choice of three features allows for a clearer graphical representation of the clustering algorithms, facilitating easier interpretation of the results. The flowchart in Figure 7 summarizes the feature selection process.

The adopted procedure for feature selection provides a comprehensive and robust approach that considers both individual importance and the interrelation among the features. After applying feature selection, machine learning algorithms were utilized to classify and separate the sources of partial discharges.

## 4. Separation and Classification of Different Sources from the Envelope

Machine learning is widely used in the separation and classification of PD signals. Both supervised and unsupervised algorithms can be applied, depending on the availability and nature of the collected data. In this paper, different methods were employed as tools to assess the signal conditioning system regarding its ability to preserve information from PD signals sufficiently for signal separation and classification. Therefore, a supervised learning method called Support Vector Machine (SVM) and the unsupervised algorithms K-means, Gaussian Mixture Model (GMM), and Mean-shift were utilized. 

The objective of the SVM algorithm is to find a hyperplane in an N-dimensional space, where N is the number of features, that distinctly classifies the data points. SVM stands out for its ability to handle high-dimensional spaces, its generalization power, and its robustness to outliers [42,43,44,45]. 

K-means is a clustering algorithm that aims to minimize the sum of squares of distances within each cluster’s outliers [42,44]. When applied to clustering different PD sources, each PD signal is assigned to the nearest cluster based on the Euclidean distance between them. GMM is a probabilistic model based on mixtures of Gaussian distributions. GMM applies a probability distribution around cluster centers to calculate the probability that a data point belongs to a particular cluster.

Mean-shift is a non-parametric clustering algorithm that identifies clusters by seeking modes or peaks in data density [44,45,46]. It does not require specifying the number of clusters beforehand, making it particularly useful when the number of clusters is unknown. Mean-shift iteratively shifts data points towards the highest density of points.

Therefore, SVM was applied to classify the PD sources. Furthermore, different clustering techniques such as K-means, GMM, and Mean-shift were used to evaluate the ability to distinguish between PD sources. The different techniques were used because the methods may perform differently on various datasets. Thus, evaluation metrics such as accuracy, precision, and recall were utilized to assess the models’ performances.

## 5. Experimental Procedures

To generate a database for the validation of the proposed system, an experimental arrangement was initially set up to measure PD signals using the method included in the international standard IEC 60270 (2000) and the proposed PD detection system. The detected PD signals were emitted from three different test objects: a PD cell with a phenolite test specimen between the electrodes, a multiple-turn coil taken from the stator of a hydrogenerator, and a dry-type potential transformer (PT) with epoxy resin insulation.

**Figure 8:**
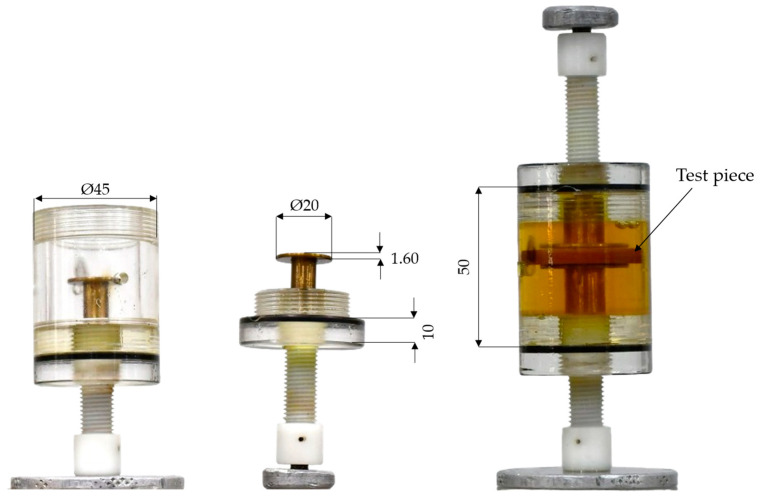
Photographs of PD cell with dimensions in millimeters.

Initially, to create a database of internal discharge in a test specimen in oil, a PD cell consisting of a cylindrical acrylic cell [47] was used. The PD cell had a pair of electrodes, one of which was connected to a high potential. The PD cell prototype was built from an acrylic cylinder, as shown in Figure 8. The electrodes used were plane, parallel, and circular, with a diameter of 20 mm and a thickness of 1.60 mm. The edges of the electrodes were semicircular in cross section to avoid the increase in electric field intensity that would occur near a sharp edge. Two nylon screws were used to define the separation of the electrodes.

The test piece, emulating a region of defective insulation, was placed in the space between the electrodes. The test piece was constructed by compressing 3 insulating plates in a layered sandwich between the two electrodes [48]. The insulation defect was made by drilling three 2 mm holes in the middle layer of the sandwich. The space around the test piece and electrodes was filled with transformer oil to avoid surface discharges and minimize corona.

**Figure 9:**
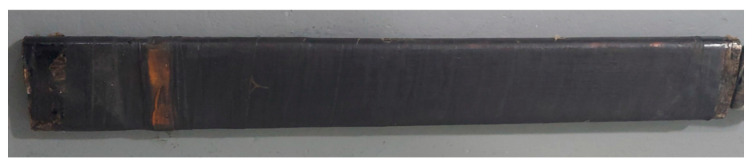
Photograph of the hydrogenerator bar.

The second test object used was a piece of multiple-turn coil taken from the stator of a hydrogenator, referred to as the hydrogenerator bar in this paper, that exhibits high magnitudes and persistence of PD. After being taken out of operation at a hydroelectric power plant, the hydrogenerator bar was donated to the laboratory for testing. Figure 9 shows a photograph of the hydrogenerator bar.

**Figure 10:**
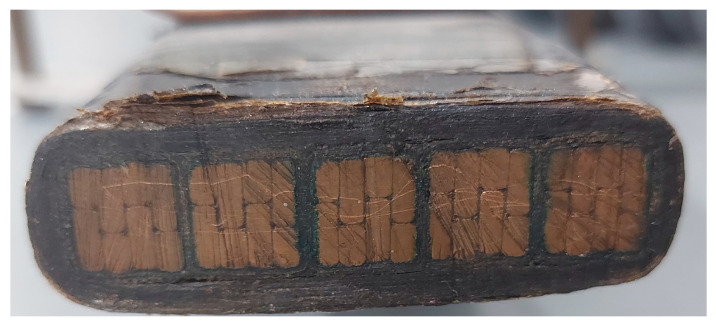
Cross-sectional view of the hydrogenerator bar.

In Figure 10, a cross-sectional view of the hydrogenerator bar can be observed. The hydrogenerator bar comprised a set of 5 turns. Each turn had 8 small copper conductors, which were also isolated from each other.

**Figure 11:**
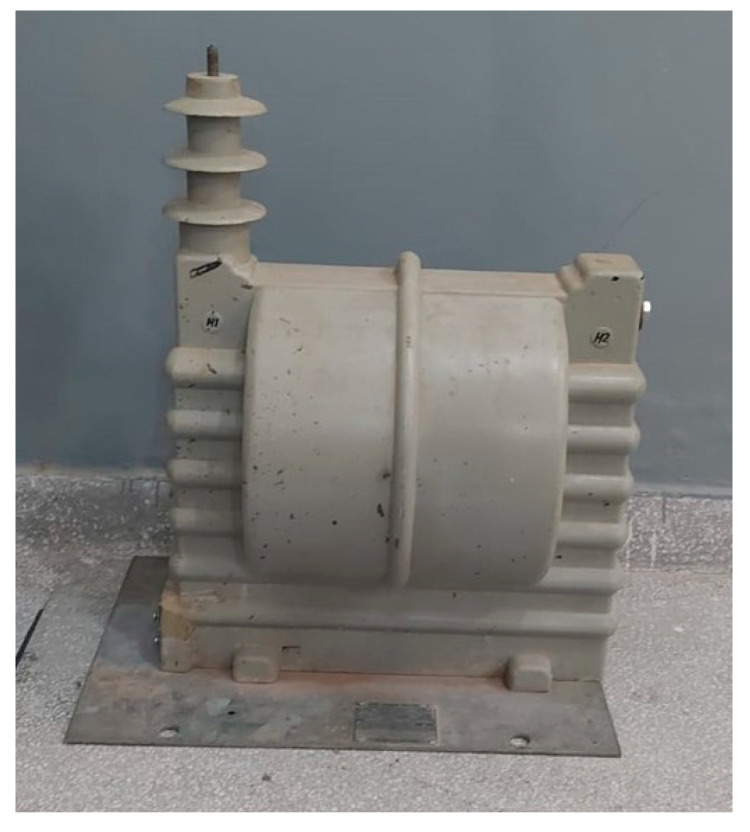
Photograph of the potential transformer.

Finally, the third test object was a dry-type PT with epoxy resin insulation and a voltage class of 36 kV, removed from operation due to internal failures, as shown in Figure 11.

**Figure 12:**
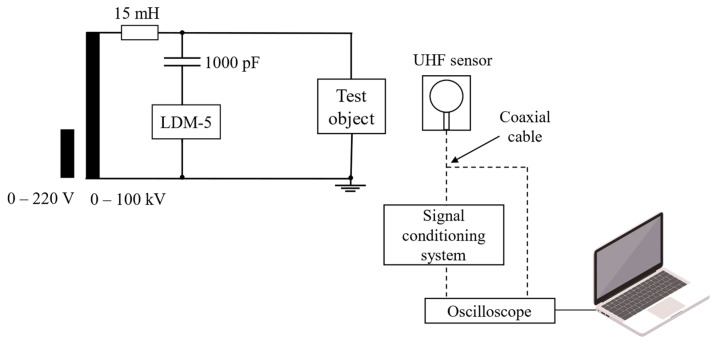
PD measurement schematic.

**Figure 13:**
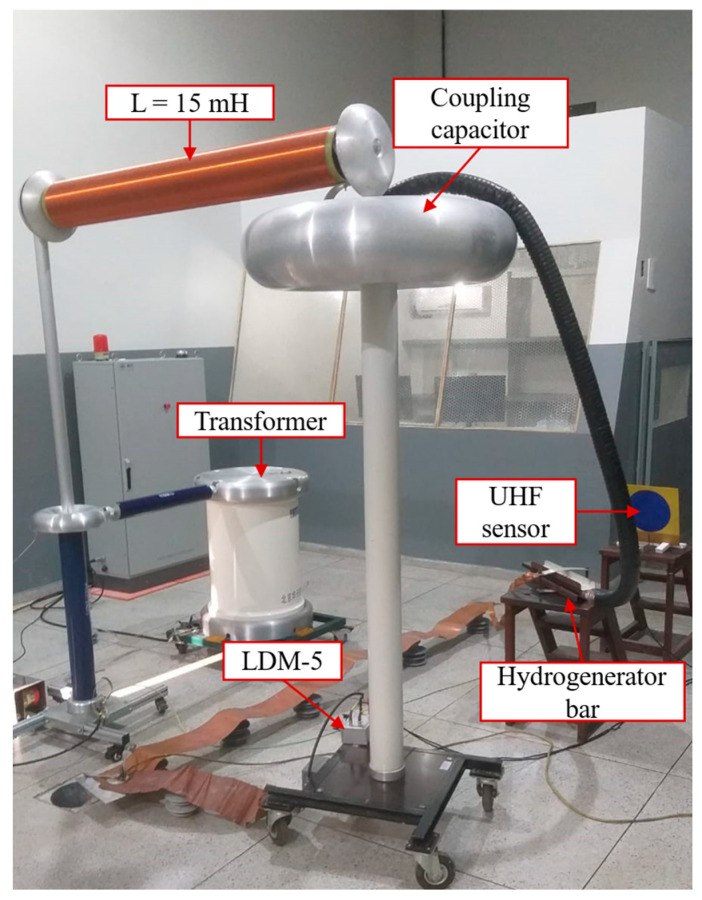
Experimental arrangement for PD measurement.

The PD measurement schematic is shown in Figure 12. The IEC 60270 (2000) method is represented by a coupling capacitor (1000 pF) and a measurement impedance (LDM-5). Additionally, a 15 mH inductor was used to filter the waveform and remove high-frequency noise from the high-voltage source. The UHF sensor used in the tests was a circular printed monopole antenna [49], considered suitable for application in the PD detection tests in view of its operating bandwidth in the range of 300~1500 MHz, and with a main value of maximum gain of approximately 4.92 dB. The photograph of the measurement arrangement can be seen in Figure 13.

The experimental procedure consisted of incrementally increasing the voltage using a transformer until PD activity was generated in the PD sources. The PD initiation voltage was 15 kV for the test cell, 17 kV for the potential transformer (PT), and 8 kV for the hydrogenerator bar. During the measurements, a background noise of approximately 5 mV was observed, significantly lower than the amplitude of the measured PD. Additionally, no external interference signals were detected.

PD pulses were acquired using an InfiniiVision DSO-X 3104A digital oscilloscope from Agilent Technologies that has a bandwidth of 1 GHz, a sampling rate of 5 GSa/s, and four channels. The PD measurements obtained were recorded simultaneously from the UHF sensor and the signal conditioning system used to smooth the radiometric PD signal.

## 6. Results and Analysis

### 6.1. Signal Conditioning System

**Figure 14:**
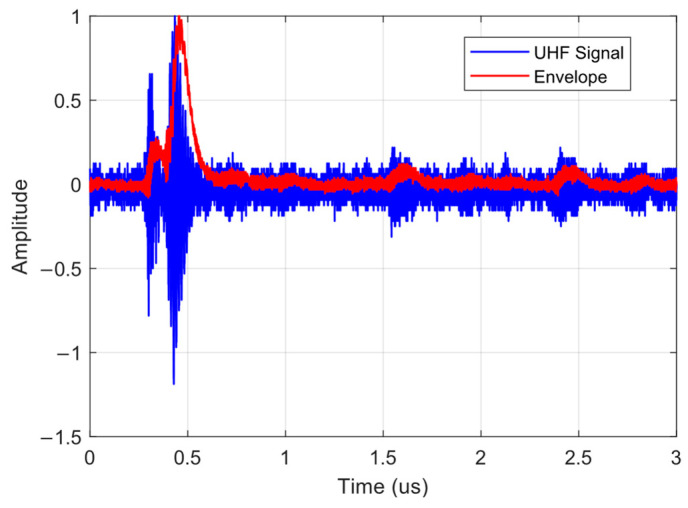
PD radiometric signal of the PT and its respective envelope.

In Figure 14, one of the radiometric PD signals of the PT and its respective envelope detected using the signal conditioning system can be observed. It is noted that the signal measured at the output of the signal conditioning system follows the envelope of the original signal, demonstrating the effectiveness of the signal conditioning system proposed.

**Figure 15:**
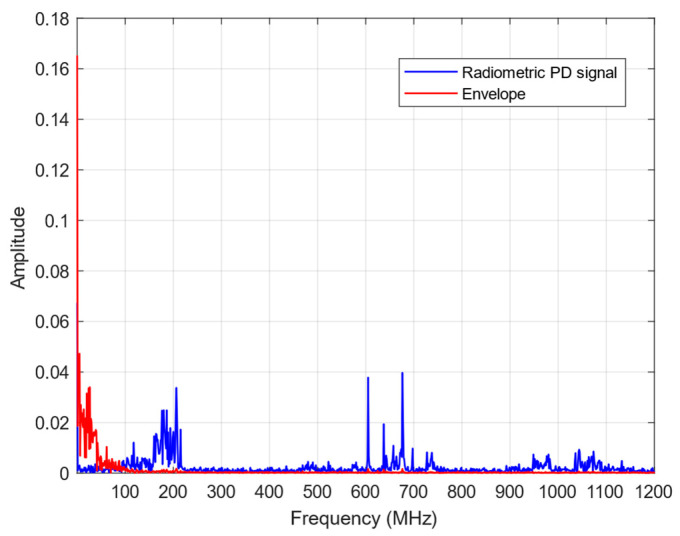
FFT response of the radiometric PD signal and its envelope.

To evaluate the frequency content of the signals before and after envelope detection, the Fast Fourier Transform (FFT) was applied to the captured radiometric PD signal from the antenna without the envelope detector, and then to the signal with the proposed signal conditioning system. Figure 15 shows the FFT response of the radiometric PD signal and its envelope.

In the graph of Figure 15, it can be observed that the high-frequency components of the signal were concentrated at up to approximately 800 MHz. However, after envelope detection, the high-frequency components of the signal are located at up to approximately 50 MHz. This indicates a reduction of around 750 MHz in the frequency level and the sampling rate requirements of the acquisition instrument.

As part of the frequency analysis, an important finding was the substantial decrease in the level of noise. This noise reduction is crucial for ensuring the accurate and reliable detection of PD signals. With lower noise levels, a higher signal-to-noise ratio can be achieved, improving the ability to differentiate and isolate the signals of interest.

### 6.2. Feature Extraction and Selection

Feature extraction was performed using an algorithm developed in an iterative software for numerical calculation. The envelopes of the PD signals from the three sources were used as the input for the algorithm, and a method for extracting and calculating basic and statistical features was implemented. 

The feature extraction, as reported in Section 3, generated a dataset with 14 columns and 2400 rows, resulting in 14 features for each set of 800 samples from a specific PD source. 

The RFE method was applied until five features were obtained. The following features were selected: average amplitude, square root amplitude, envelope time, rise time, and fall time. From the application of the mutual information algorithm, the following features were selected: average amplitude, energy, envelope time, rise time, and fall time. 

The six features defined by the union of features selected by RFE and mutual information were analyzed for their correlation. Table 1 shows the correlation coefficient matrix.

After analyzing the correlation between the features selected by RFE and mutual information, it was found that the following features had the lowest correlation between them: square root amplitude, energy, and fall time. In the next section, the selected features will be evaluated using SVM, K-means, GMM, and Mean-shift.

### 6.3. Separation and Classification of Envelopes

To validate whether the selected features could characterize the different PD sources, the supervised SVM method was applied. The database was partitioned as follows: 30% of the samples were used for testing and 70% for training. The effectiveness of the method was evaluated using the average accuracy, precision, recall and explicitly detailed in the confusion matrix, as shown in Table 2.

From the confusion matrix, it can be inferred that the SVM model achieved an accuracy rate of 100% for the hydrogenerator bar, 80% for the PT, and 97.0% for the PD cell. In Table 2, the term “Accuracy” represents the general performance of the model, indicating the proportion of correct predictions made by the model in comparison to the total number of predictions made. The average accuracy of the SVM model was 0.92, indicating a strong performance of the model. 

The recall and precision values are also provided for each class. Precision measures the accuracy of positive predictions made by the model. The precision for the hydrogenerator bar is 1, indicating no false positives in the predictions made by the model. The precision for the PT was 0.96, indicating that 96% of instances identified as the PT by the model were correct, while for the PD cell, the precision was 0.830, indicating 83% precision in identifying instances as the PD cell. Recall measures the proportion of true positive instances correctly identified by the model out of all actual positive instances. For the hydrogenerator bar, all positive instances were correctly identified, resulting in a recall of 1. For the PT, the model identified 80% of true positive instances, yielding a recall of 0.80. Similarly, for the PD cell, the model achieved a recall of 0.97, correctly identifying 97% of positive instances. 

**Figure 16:**
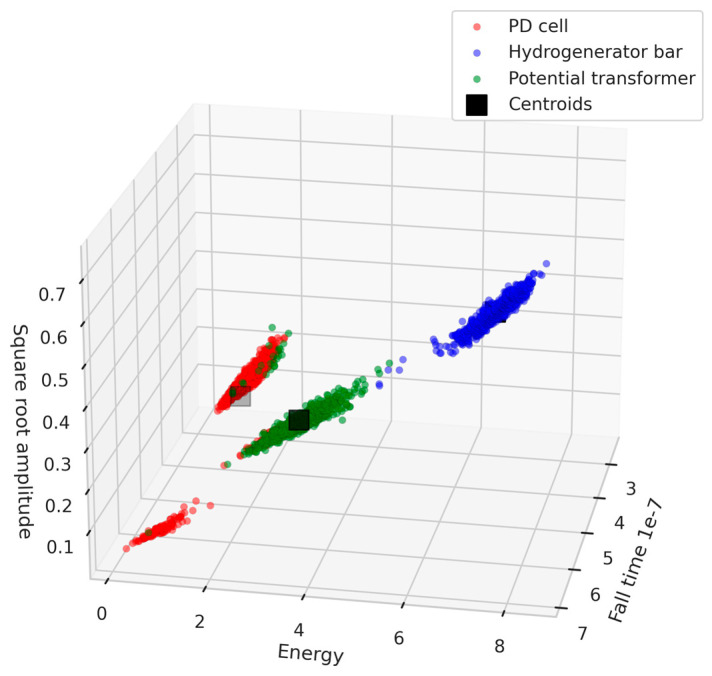
Three-dimensional scatter plot of the K-means algorithm.

**Figure 17:**
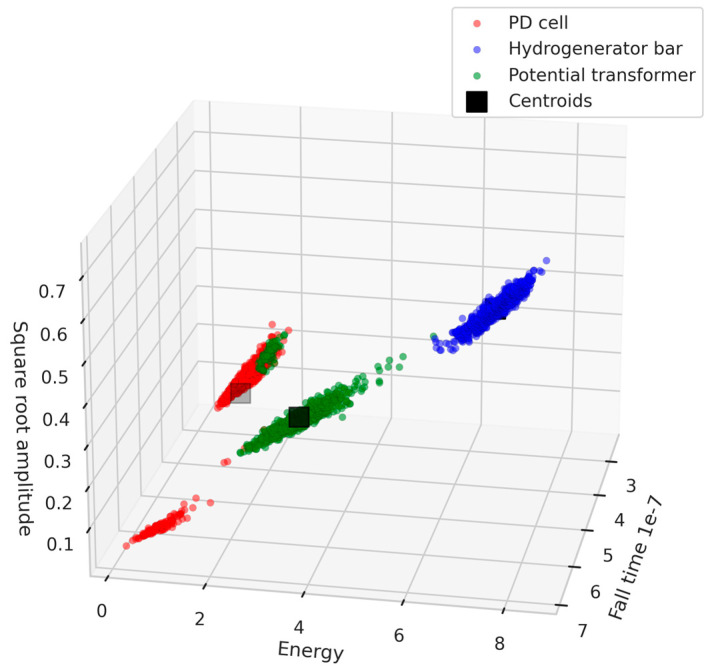
Three-dimensional scatter plot of the GMM algorithm.

**Figure 18:**
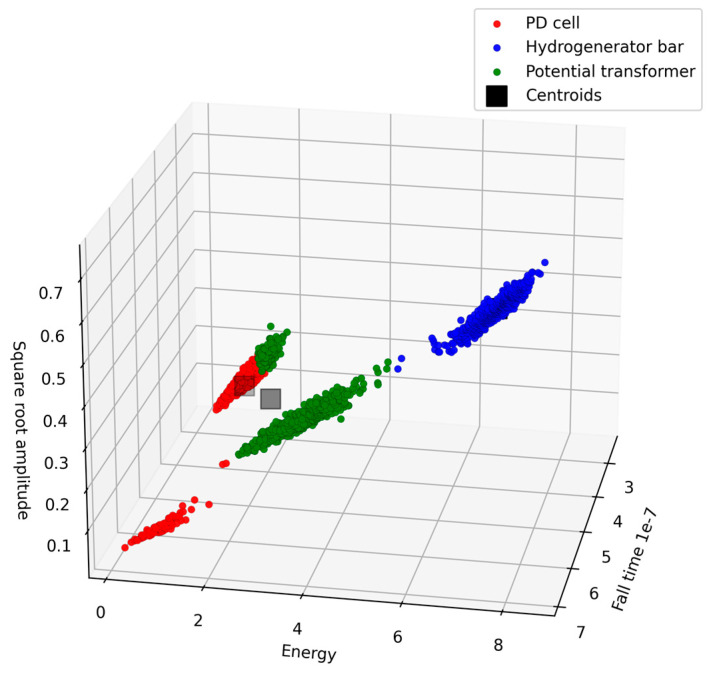
Three-dimensional scatter plot of the Mean-shift algorithm.

The K-means, GMM, and Mean-shift clustering algorithms were used to separate the different test objects. Figure 16, Figure 17 and Figure 18 show the 3D scatter plots of the K-means, GMM, and Mean-shift algorithms. 

The three clustering algorithms employed successfully separated the three test objects. To identify the algorithm with the highest accuracy rate, a comparative analysis of algorithm performance was conducted based on the confusion matrix. In this process, 70% of the data from each class were utilized for the initial determination of centroids, and the remaining 30% were used for testing purposes. Table 3, Table 4 and Table 5 show the confusion matrices of the K-means, GMM, and Mean-shift algorithms, respectively.

Table 3, Table 4 and Table 5 provide an overview of the performance of the three clustering algorithms: K-means, GMM, and Mean-shift. For K-means, all classes achieved high precision values (1 for the hydrogenerator bar, 0.94 for the PT, and 0.85 for the PD cell), with recall values of 1, 0.83, and 0.95, respectively. Similarly, the GMM model exhibited consistent accuracy, with precisions of 1, 0.92, and 0.86 and recalls of 1, 0.84, and 0.92 for each class. The overall accuracy of the GMM model was 0.93. Lastly, the Mean-shift algorithm obtained a slightly lower average accuracy value of 0.90, with precisions of 0.99, 0.86, and 0.85, and achieved recalls of 1, 0.85, and 0.85 for each class. It is noteworthy that the Mean-shift algorithm has the capability of automatically identifying the number of clusters, representing a significant advantage, especially in practical field applications. Table 6 presents a comprehensive overview of the evaluation metrics—accuracy, precision, and recall—for the four models considered in this study: SVM, K-means, GMM, and Mean-shift.

Overall, the three algorithms demonstrated satisfactory accuracy values. Therefore, the proposed signal conditioning system proved capable of reducing the required sampling rate by the acquisition system while maintaining sufficient information for the separation and classification of radiometric signals.

## 7. Discussion

This research focuses on two challenges: reducing the hardware requirements for radiometric partial discharge detection in substations and separating partial discharge sources for accurate signal interpretation.

The first solution involves the development of a signal conditioning system based on the envelope detection technique. The results presented in Figure 14 demonstrate the successful application of the signal conditioning system, where the envelope of the PD signal follows the original signal. The frequency analysis, as shown in Figure 15, highlights a significant reduction in high-frequency components up to 50 MHz after envelope detection, thereby reducing the sampling rate requirements. This reduction not only enhances the efficiency of the acquisition system but also contributes to noise reduction due to the use of the RF amplifier, a critical factor for accurate PD signal detection.

The second proposed solution utilizes machine learning techniques for signal separation based on features extracted from the signal envelope. The feature extraction process, discussed in Section 6.2, resulted in a dataset with the selected features of square root amplitude, energy, and fall time. 

The SVM method for classification achieved high accuracy rates, as evidenced by the confusion matrix in Table 2. The SVM model demonstrated high precision, particularly in classifying the hydrogenerator bar and PT sources.

Furthermore, the application of clustering algorithms (K-means, GMM, and Mean-shift) for separation of PD sources showcased promising results. The 3D scatter plots in Figure 16, Figure 17 and Figure 18 illustrate the successful separation of test objects. The comparative analysis of algorithm performance, as presented in Table 6, indicates high accuracy rates for K-means and GMM, both achieving an accuracy value of 0.93.

The outcomes of this study have significant implications for the field of PD detection and classification in substation environments. The successful implementation of the signal conditioning system and the machine learning-based signal separation approach opens avenues for more economical, scalable, and reliable PD monitoring systems. The reduction in hardware requirements and the improved signal-to-noise ratio contribute to the feasibility of real-time PD detection in challenging substation conditions.

In future work, the developed signal conditioning system will be evaluated with more test objects to ensure the effectiveness of the system. Furthermore, the system will be applied and evaluated in the location of the PD sources. The combination of the proposed techniques with a radiometric method focusing solely on the received signal strength information is promising for increasing the accuracy and efficiency of identifying and locating PD sources within the substation, with the absence of synchronization between sensors and substantial reduction in data transmission and storage.

## 8. Conclusions

This paper developed and evaluated a signal conditioning system based on the envelope detection technique. The signal conditioning system proved to be efficient in reducing the hardware requirements of the acquisition system, resulting in a significant decrease in the frequency of detected signals, with the main signal components concentrated at up to about 50 MHz. This frequency level makes it unnecessary to use instruments with high sampling rates, thus making feasible the monitoring of PD using the radiometric method in substations.

The proposed signal conditioning system is versatile, serving the purposes of detecting, localizing, and classifying PD. This study specifically focused on its application in separating and classifying PD signals, aiming to enhance the PD identification process. Signals were successfully separated and classified based on features extracted and selected from the envelopes using the developed algorithm.

The efficacy of the conditioning system was validated through the application of machine learning models, showcasing its capability in classifying and separating partial discharge sources based on information extracted from the signal envelope. The summary of the results of the metrics used to evaluate the models (Table 6) shows that the applied models had good recall, precision, and accuracy results, with accuracy values of 0.92 for SVM, 0.93 for K-means and GMM, and 0.90 for Mean-shift, thus confirming the effectiveness of the system in separating and classifying PD signals.

In conclusion, the presented solutions offer advances in the field of PD detection and classification, offering potential applications in substation maintenance and monitoring. Further research and development in this direction can contribute to the enhancement of power system reliability and the prevention of equipment failures related to partial discharges.

## Figures and Tables

**Table 1:** Correlation coefficient matrix.

	$A_{a}$	$A_{r2}$	$E_{s}$	$T_{t}$	$T_{r}$	$T_{f}$
$A_{a}$	1	0.64	−0.05	0.03	0.31	0.01
$A_{r2}$	0.64	1	0.02	−0.01	0.14	−0.01
$E_{s}$	−0.05	0.02	1	−0.34	−0.70	−0.32
$T_{t}$	0.03	0.01	−0.34	1	0.20	1.0
$T_{r}$	0.31	0.14	−0.70	0.20	1	0.16
$T_{f}$	0.01	−0.01	−0.31	0.99	0.16	1

**Table 2:** Confusion matrix, precision, recall and average accuracy for the SVM model.

Predicted Label					
**True Label**		**Hydrogenerator Bar**	**PT**	**PD Cell**	**Recall**
	Hydrogenerator bar	**240**	**0**	**0**	1
	PT	**0**	**192**	**48**	0.80
	PD cell	**0**	**7**	**233**	0.97
	**Precision**	1	0.96	0.830	
**Accuracy** = 0.92					

**Table 3:** Confusion matrix, precision, recall and average accuracy for K-means.

Predicted Label					
**True Label**		**Hydrogenerator Bar**	**PT**	**PD Cell**	**Recall**
	Hydrogenerator bar	**240**	**0**	**0**	1
	PT	**0**	**200**	**40**	0.83
	PD cell	**0**	**12**	**228**	0.95
	**Precision**	1	0.94	0.85	
**Accuracy** = 0.93					

**Table 4:** Confusion matrix, precision, recall and average accuracy for the GMM.

Predicted Label					
**True Label**		**Hydrogenerator Bar**	**PT**	**PD Cell**	**Recall**
	Hydrogenerator bar	**240**	**0**	**0**	1
	PT	**0**	**203**	**37**	0.84
	PD cell	**0**	**17**	**223**	0.92
	**Precision**	1	0.92	0.86	
**Accuracy** = 0.93					

**Table 5:** Confusion matrix, precision, recall and average accuracy for the Mean-shift.

Predicted Label					
**True Label**		**Hydrogenerator Bar**	**PT**	**PD Cell**	**Recall**
	Hydrogenerator bar	**240**	**0**	**0**	1
	PT	**1**	**205**	**34**	0.85
	PD cell	**0**	**34**	**206**	0.85
	**Precision**	0.99	0.86	0.85	
**Accuracy** = 0.90					

**Table 6:** Evaluation metrics of the models.

	Accuracy	Precision			Recall		
		H. Bar *	PT	PD Cell	H. Bar *	PT	PD Cell
**SVM**	0.92	1	0.96	0.83	1	0.80	0.97
**K-means**	0.93	1	0.94	0.85	1	0.83	0.95
**GMM**	0.93	1	0.92	0.86	1	0.84	0.92
**Mean-shift**	0.90	0.99	0.86	0.85	1	0.85	0.85

* H. bar = hydrogenerator bar.

## Data Availability

The data presented in this study are available on request from the corresponding author.
